# Atmospheric deposition of chlorinated and brominated polycyclic aromatic hydrocarbons in central Europe analyzed by GC-MS/MS

**DOI:** 10.1007/s11356-021-15038-3

**Published:** 2021-06-26

**Authors:** Rong Jin, Benjamin A. Musa Bandowe, Minghui Zheng, Guorui Liu, Barbora Nežiková, Roman Prokeš, Pavel Čupr, Jana Klánová, Gerhard Lammel

**Affiliations:** 1 School of Environment, Hangzhou Institute for Advanced Study, University of Chinese Academy of Sciences, Hangzhou, 310024 China; 2 Multiphase Chemistry Department, Max-Planck Institute for Chemistry, Hahn-Meitner-Weg 1, 55128 Mainz, Germany; 3 State Key Laboratory of Environmental Chemistry and Ecotoxicology, Research Centre for Eco-environmental Sciences, Chinese Academy of Sciences, Beijing, 100085 China; 4 Research Centre for Toxic Compounds in the Environment, Masaryk University, Kamenice 5, 62500 Brno, Czech Republic

**Keywords:** Halogenated PAHs, Triple quadrupole mass spectrometry, Analytical method, Total deposition, Wet and dry deposition

## Abstract

Chlorinated and brominated polycyclic aromatic hydrocarbons (ClPAHs and BrPAHs) are persistent organic pollutants that are ubiquitous in the atmospheric environment. The sources, fate, and sinks in the atmosphere of these substances are largely unknown. One of the reasons is the lack of widely accessible analytical instrumentation. In this study, a new analytical method for ClPAHs and BrPAHs using gas-chromatography coupled with triple quadrupole mass spectrometry is presented. The method was applied to determine ClPAHs and BrPAHs in total deposition samples collected at two sites in central Europe. Deposition fluxes of ClPAHs and BrPAHs ranged 580 (272–962) and 494 (161–936) pg m^−2^ day^−1^, respectively, at a regional background site, Košetice, and 547 (351–724) and 449 (202–758) pg m^−2^ day^−1^, respectively, at a semi-urban site, Praha-Libuš. These fluxes are similar to those of PCBs and more than 2 orders of magnitude lower than those of the parent PAHs in the region. Seasonal variations of the deposition fluxes of these halogenated PAHs were found with maxima in summer and autumn, and minima in winter at Košetice, but vice versa at Praha-Libuš. The distribution of ClPAHs and BrPAHs between the particulate and dissolved phases in deposition samples suggests higher degradability of particulate BrFlt/Pyr and BrBaA than of the corresponding ClPAHs. A number of congeners were detected for the first time in the atmospheric environment.

**Supplementary Information:**

The online version contains supplementary material available at 10.1007/s11356-021-15038-3.

## Introduction

Polycyclic aromatic hydrocarbons (PAHs) are globally ubiquitous pollutants drawing a lot of attention as posing a risk for human health upon inhalation exposure, and for ecosystems upon atmospheric deposition. Accordingly, PAHs are regulated under the auspices of international conventions for the protection of the environment (OSPAR 2019; UNECE 1998). Chlorinated and brominated polycyclic aromatic hydrocarbons (XPAHs; ClPAHs and BrPAHs) are halogenated derivatives of PAHs. Apart from the polychlorinated naphthalenes (PCN), they were only recently addressed in the environment and with some of them identified to be more toxic than their corresponding parent PAH congeners (Ohura et al. 2007; Huang et al. 2018). The related environmental risks might be comparable to those of PCDD/Fs (Ohura et al. 2007; Jin et al. 2020b). Previous studies have identified XPAHs in many environmental matrices, such as air (Jin et al. 2017a; Jin et al. 2017c) and soil (Ieda et al. 2011, Jin et al. 2020a), suggesting that XPAHs are ubiquitous in the environment (Sun et al. 2013; Jin et al. 2020b). Vapor pressures of mono- and di-XPAHs are at the range of 10^−7^ to 10^−2^ Pa, indicating that the XPAHs are semi-volatile organic compounds (SVOCs), potentially undergoing long-range atmospheric transport (Goldfarb and Suuberg 2008; Fu and Suuberg 2012; Jin et al. 2020a). Therefore, XPAHs are classified as persistent organic pollutants (POPs). However, the knowledge of XPAHs’ environmental occurrence and fate characteristics are still very limited. Atmospheric XPAHs so far had mainly been studied in East Asian countries, i.e., China and Japan (Ohura et al. 2005; Ohura et al. 2013; Jin et al. 2017a). Their occurrence in other regions, such as central Europe, where their emission sources might be related to different industries and consumption patterns still remain unknown.

Lack of a standardized analytical method is one of the main reasons for lack of XPAH studies. Potentially, there could be thousands of XPAH isomers, making their separation and qualification in environmental matrices difficult. In addition, XPAH concentrations in many environmental matrices were reported at ultra-trace level, such as 2–3 orders of magnitude lower than PAHs in air samples (Ohura et al. 2009; Jin et al. 2017a). Such low concentrations render them not easily quantifiable by many widely available GC-MS instruments (that are commonly used for analysis of other PAHs) due to challenges with analytical sensitivity. Gas chromatography coupled with triple quadrupole mass spectrometry (GC-MS/MS) has been applied as an alternative to GC, coupled with high-resolution mass spectrometry (GC-HRMS) which is widely used for the analysis of POPs, namely polychlorinated dibenzo-*p*-dioxins and dibenzofurans (PCDD/Fs), polychlorinated biphenyls (PCBs), and polychlorinated naphthalenes (PCNs) (Banerjee et al. 2012; Li et al. 2014; van Bavel et al. 2015; Wu et al. 2017). In these studies, GC-MS/MS could satisfy the methodological requirements for determination of POPs in many environmental matrices (e.g., air, soil, and human milk). GC-MS/MS instruments might therefore be an alternative analytical instrument (to the GC-HRMS) for the analysis of XPAHs in environmental samples. The development of an analytical method for XPAH on GC-MS/MS would also provide the potential for further applications and method comparison.

Marine and terrestrial ecosystems are exposed to POPs via atmospheric depositions (Mi 2012; Nežiková et al. 2019). Deposition limits atmospheric residence time, and in combination with re-volatilization from soils and water bodies shapes the long-range transport potential of many POPs. Deposition fluxes for many POPs, such as PAHs, organochlorine pesticides (OCPs), and PCBs, have been measured and reported (Atlas and Giam 1988; Bidleman 1988; Škrdlíková et al. 2011; Zhang et al. 2012; Mi 2012; Jiménez et al. 2015; Nežiková et al. 2019). Previous studies reported that deposition of lipophilic organics in air is dominated by dry particle deposition (Mi 2012; Luo et al. 2014; Luo et al. 2016). XPAHs have been detected in both the gaseous and particulate phases (Jin et al. 2017a and 2017c). Similar to other lipophilic organic compounds, XPAH particulate mass fraction tends to increase with decreasing vapor pressure (Jin et al. 2017c). For some 5-ring XPAHs, such as 6-ClBaP, particulate mass fractions could exceed 80% (Jin et al. 2017c). In addition, in heavily polluted atmospheric environments, such as during haze, particulate mass fractions will increase significantly (Jin et al. 2017c). This underpins the significance of the pollutant’s deposition for receiving ecosystems and foodwebs. Despite the relevance of this “emerging” class of pollutants and the potential adverse effects on health and ecosystems, XPAHs have not yet been studied in the atmospheric environment in Europe.

The aim of this study was to develop a sensitive GC-MS/MS method for XPAHs in bulk deposition samples, and to apply the method to first time quantify XPAH in atmospheric deposition samples, hence, address exposure of terrestrial ecosystems to XPAHs.

## Methods and materials

### Chemicals

There were 20 ClPAH congeners and 21 BrPAH congeners analyzed in this study (Table 1). Standards of XPAH congeners were purchased from commercial laboratories, i.e., 9,10-dichloroanthracene (9,10-Cl_2_Ant), 2,6-dibromoanthracene (2,6-Br_2_Ant), 2,7-dibromophenanthrene (2,7-Br_2_Phe), 1,6-dibromopyrene (1,6-Br_2_Pyr), 7-bromobenz[a]anthracene (7-BrBaA), 4-bromobenz[a]anthracene (4-BrBaA), 3-bromofluoranthene (3-BrFlt), and 1,8-dibromopyrene (1,8-Br_2_Pyr) were purchased from Tokyo Chemistry Industry (TCI), Japan; 7-chlorobenz[a]anthracene (7-ClBaA), 7,12-dichlorobenz[a]anthracene (7,12-Cl_2_BaA), and ^13^C_6_-labelled 7,12-Cl_2_BaA were purchased from Cambridge Isotope Laboratories, Tewksbury, USA; 1,4-dichloroanthracene (1,4-Cl_2_Ant), 1,5-dichloroanthracene (1,5-Cl_2_Ant), 2,3-dichloroanthracene (2,3-Cl_2_Ant), 9,10-dibromoanthracene (9,10-Br_2_Ant), 3-bromophenanthrene (3-BrPhe), 9,10-dibromophenanthrene (9,10-Br_2_Phe), and 1-bromopyrene (1-BrPyr) were purchased from Sigma Aldrich, St. Louis, USA; 9-chlorophenanthrene (9-ClPhe), 2-chloroanthracene (2-ClAnt), ^13^C_6_-labelled 2-ClAnt, 9,10-dichlorophenanthrene (9,10-Cl_2_Phe), 1,8-dichloropyrene (1,8-Cl_2_Pyr), 2-bromoanthracene (2-BrAnt), 9-bromophenanthrene (9-BrPhe), 9-BrPhe-d_9_, 2,3-dibromoanthracene (2,3-Br_2_Ant), 2,6-dibromoanthracene (2,6-Br_2_Ant), and 6-bromobenzo[a]pyrene (6-BrBaP) were purchased from Toronto Research Chemicals, Canada; 3-chlorofluoranthene (3-ClFlt) and 6-chlorobenzo[a]pyrene (6-ClBaP) were purchased from Campro Scientific, Berlin, Germany.

**Table 1:** Retention time, quantification, and qualification masses and energies of XPAH congeners.

Name	Abbreviation	Retention time	Quantification ions	Qualification ions	Energy
3-Chlorophenanthrene	3-ClPhe	26.1	212.0→176.1	214.0→176.1	30
9-Chlorophenanthrene/2-chlorophenanthrene	9-/2-ClPhe	26.6	212.0→176.1	214.0→176.1	30
1-Chloroanthracene	1-ClAnt	27.2	212.0→176.1	214.0→176.1	30
2-Chloroanthracene	2-ClAnt	27.3	212.0→176.1	214.0→176.1	30
9-Chloroanthracene	9-ClAnt	27.5	212.0→176.1	214.0→176.1	30
3-Bromophenanthrene	3-BrPhe	33.4	258.0→176.1	256.0→176.1	30
9-Bromophenanthrene	9-BrPhe	34.2	258.0→176.1	256.0→176.1	30
2-Bromophenanthrene	2-BrPhe	34.5	258.0→176.1	256.0→176.1	30
1-Bromoanthracene	1-BrAnt	35.4	258.0→176.1	256.0→176.1	30
9-Bromoanthracene	9-BrAnt	35.4	258.0→176.1	256.0→176.1	30
2-Bromoanthracene	2-BrAnt	36.2	258.0→176.1	256.0→176.1	30
1,4-Dichloroanthracene	1,4-Cl_2_Ant	40.6	246.0→176.1	248.0→176.1	30
1,5-Dichloroanthracene/9,10-dichloroanthracene	1,5-/9,10-Cl_2_Ant	41.3	246.0→176.1	248.0→176.1	30
9,10-Dichlorophenanthrene	9,10-Cl_2_Phe	42.0	246.0→176.1	248.0→176.1	30
2,3-Dichloroanthracene	2,3-Cl_2_Ant	43.2	246.0→176.1	248.0→176.1	30
3-Chlorofluoranthene	3-ClFlt	44.4	236.0→200.1	238.0→200.1	35
4-Chloropyrene	4-ClPyr	46.2	236.0→200.1	238.0→200.1	35
1-Chloropyrene	1-ClPyr	46.2	236.0→200.1	238.0→200.1	35
3-Bromofluoranthene	3-BrFlt	47.6	280.0→200.1	282.0→200.1	40
1,8-Dibromoanthracene/1,5-dibromoanthracene	1,8-/1,5-Br_2_Ant	48.3	335.9→176.1	333.9→176.1	30
9,10-Dibromoanthracene	9,10-Br_2_Ant	48.5	335.9→176.1	333.9→176.1	30
2,7-Dibromophenanthrene	2,7-Br_2_Phe	48.6	335.9→176.1	333.9→176.1	30
4-Bromopyrene	4-BrPyr	48.8	335.9→176.1	333.9→176.1	30
9,10-Dibromophenanthrene	9,10-Br_2_Phe	48.9	280.0→200.1	282.0→200.1	40
1-Bromopyrene	1-BrPyr	48.9	280.0→200.1	282.0→200.1	40
2,6-Dibromoanthracene	2,6-Br_2_Ant	49.0	335.9→176.1	333.9→176.1	30
2,3-Dibromoanthracene	2,3-Br_2_Ant	49.4	335.9→176.1	333.9→176.1	30
3,8-Dichlorofluoranthene	3,8-Cl_2_Flt	49.6	270.0→200.1	272.0→200.1	35
1,6-Dichloropyrene/1,8-dichloropyrene	1,6-/1,8-Cl_2_Pyr	50.6	270.0→200.1	272.0→200.1	35
7-Chlorobenz[a]anthracene	7-ClBaA	52.7	262.1→226.1	264.1→226.1	30
1,6-Dibromopyrene/1,8-dibromopyrene	1,6-/1,8-Br2Pyr	54.8	359.9→200.1	357.9→200.1	40
7-Bromobenz[a]anthracene/4-bromobenz[a]anthracene	7-/4-BrBaA	54.8	306.0→226.1	308.0→226.1	40
7,12-Dichlorobenz[a]anthracene	7,12-Cl_2_BaA	56.0	296.0→226.1	298.0→226.1	30
6-Chlorobenzo[a]pyrene	6-ClBaP	60.7	286.1→250.1	288.1→250.1	35
6-Bromobenzo[a]pyrene	6-BrBaP	64.1	332.1→250.1	330.1→250.1	35
Internal standards					
2-Chloroanthracene-^13^C_6_	2-ClAnt-^13^C	27.3	218.1→182.1	220.1→182.1	30
7-Chlorobenz[a]anthracene-^13^C_6_	7-ClBaA-^13^C	52.7	268.1→232.1	270.1→232.1	30
Recovery standards					
9-Bromophenanthrene-d_9_	9-BrPhe-d_9_	33.6	265.1→184.1	267.1→184.1	30

### Sampling

Total deposition samples were collected at the Czech Hydrometeorological Institute’s (CHMI) National Atmospheric Observatory, Košetice (KOS; 49.56° N/15.12° E) and the CHMI station Praha-Libuš (PRA; 50.01° N/14.46° E) in the Czech Republic. KOS is a regional background site of central Europe, while PRA is a semi-urban site in immediate vicinity of forests and 10 km south of the city center of Prague. Samples were collected every 3 months from July 2013 to August 2015 in Košetice (9 samples) and from October 2013 to August 2015 in Praha-Libuš (8 samples). Sampling time series are shown in Table S1.

Details of the deposition sampler were described in our previous study (Nežiková et al. 2019). The deposition sampler is based on a sampler successfully applied in the MONARPOP project (Jakobi et al. 2015; Offenthaler et al. 2009), and improved later (Čupr and Pěnkava 2012; Nežiková et al. 2019). It consists of a collection funnel (250 mm diameter) and a stainless-steel particulate filter holder at the bottom. Particles are collected on a glass microfiber filter (GFF, 70 mm, Whatman, USA) and the dissolved phase of depositions is collected on a XAD sorbent. Filters and sorbents were extracted by dichloromethane (DCM, 3 cycles) and dried before usage. To this end, XAD-2 sorbent (Supelco, USA) is put into a glass column connected to the base of the filter holder. For sample collection, the funnel was not rinsed. After sampling, all the samples were stored in the fridge with the temperature < −18 °C until analysis.

### Sample analysis

Before pretreatment, GFF and XAD-2 sorbent were taken out to attain room temperature, and then spiked with 1 ng internal standard consisting of ^13^C_6_-2-ClAnt and ^13^C_6_-7,12-Cl_2_BaA. After that, the XAD-2 sorbents were extracted by DCM for 3 cycles with each cycle of 45 min duration using Soxhlet. The filters were extracted by DCM through shaking 3 cycles of 2 min each, 2000 spins min^−1^ (vortex mixer Stuart Scientific SA7, Stone, UK). The extracts were concentrated, and transferred into  commercial (containing  500 mg active silica gel), then eluted with 10 mL mixed solvents (hexane:DCM=4:1 V/V), followed by 10 mL DCM. The eluates from each sample were combined and evaporated with a gentle stream of nitrogen to a volume of  ≈500 μL. Each concentrated extract was then spiked with the recovery standard (9-BrPhe-d_9_), transferred into a GC-vial, ready for GC-MS/MS measurements.

XPAH congeners were analyzed by GC-MS/MS using a Trace 1310 gas chromatograph interfaced to a TSQ 8000 EVO triple quadrupole mass selective detector (Thermo Scientific, Waltham, USA). The separation was conducted on a DB-5 MS capillary column (60m×0.25mm×0.25 μm, Agilent Technologies, Waldbronn, Germany). The initial temperature was 50 °C, then increased to 175 °C with a rate of 25 °C min^−1^, then increased to 200 °C with a rate of 5 °C min^−1^, stayed for 28 min, then heated to 300 °C with a rate of 8 °C min^−1^, and finally to 310 °C with a rate of 1 °C min^−1^. The inlet, transfer line, and ion source temperatures were 280, 290, and 250 °C, respectively. Data was acquired under multireaction monitoring (MRM) mode.

## Results and discussion

### Validation of the pretreatment method

The clean-up processes were performed using commercial SPE cartridges containing 500 mg active silica gel and the solvents ethyl acetate (EA), DCM and hexane, previously used for analyzing nitrated and oxygenated PAHs (Degrendele et al. 2016; Lammel et al. 2017; Nežiková et al. 2019; Shahpoury et al. 2018). In order to optimize efficiency and completeness of the clean-up processes, we tested three different sequences of 3 elutions using 10 mL EA, 10 mL DCM, and 10 mL hexane:DCM=4:1 mixture (Table S2). XPAH mixtures of known amount (1 ng) were transferred onto the SPE columns and sequentially eluted as previously described. Each elute was collected and analyzed separately. It is found that more than 95% of the amount of targeted XPAHs was eluted by the 10 mL Hex:DCM=4:1 mixture alone. Another 10 mL DCM was added to make sure that as much XPAH congeners as possible could be eluted.

**Figure. 1:**
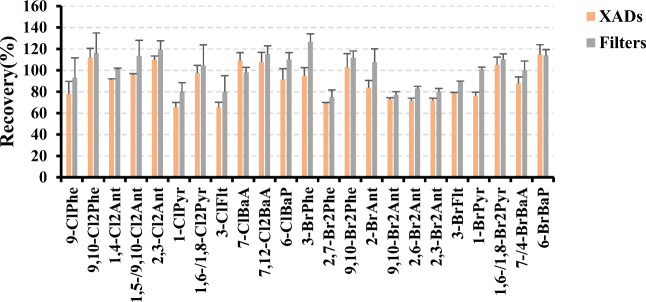
Recoveries (%) of XPAH congeners after the clean-up process. Error bars indicate the standard deviation of replicate (n = 3) experiments.

In order to test the recoveries of XPAHs in the real sample matrices after the whole pretreatment processes (including extraction), we added 20 pg of XPAH congener standard to 3 cleaned filters and 3 XADs, extracted and purified the samples. Internal standards and recovery standards were added to the cleaned extracts before injection. The recoveries of individual XPAH congeners ranged from 65 to 115% for XAD samples, and from 75 to 126% for filter samples (Figure 1). These ranges met the requirement for POPs analysis by using the isotopic GC-HRMS method (United States Environmental Protection Agency 2008, 2010). The procedure was controlled by lab blanks. To this end, cleaned filters (n=3) and XAD sorbents (n=3) were spiked with internal standards, extracted, and cleaned up with the same procedures and solvents as applied for the real samples and spiked filters/XAD. The concentrations of all XPAH congeners in these blanks were below the detection limits (see below).

### Validation of the instrumental method

The GC oven temperature program was set according to our previous studies (Jin et al. 2017b). Even though the targeted XPAH congeners in this study were not consistent with previous studies, 20 ClPAH and 21 BrPAH congeners could be quantified well within 66 min. Several XPAH congeners could not be separated from each other, i.e., 9-ClPhe and 2-ClPhe, 1,5-Cl_2_Ant and 9,10-Cl_2_Ant, 1,8-Br_2_Ant and 1,5-Br_2_Ant, 1,6-Cl_2_Pyr and 1,8-Cl_2_Pyr, 1,6-Br_2_Pyr and 1,8-Br_2_Pyr, 7-BrBaA and 4-BrBaA, and, therefore, were quantified together. In addition, 6 XPAH congeners and one pair of not separated congeners, i.e., 2,3-Cl_2_Ant, 4-ClPyr, 2,7-Br_2_Phe, 2,3-Br_2_Ant, 1,8-Br_2_Pyr, 4-BrBaA, and 1,6-/1,8-Cl_2_Pyr, were detected for the first time. The retention times of the targeted XPAH congeners are listed in Table 1.

**Figure. 2:**
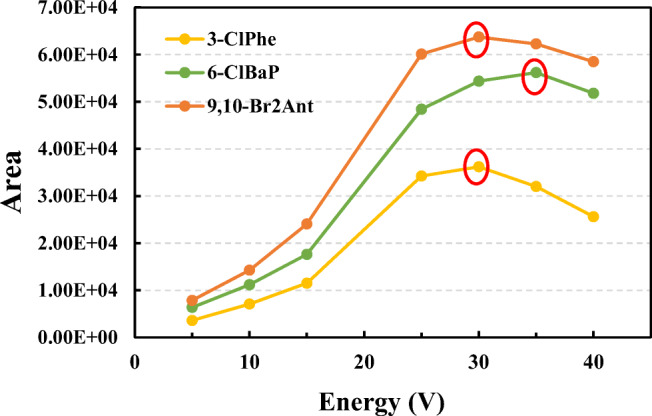
Examples of the collision energy selection process for XPAHs. The energy under which the peak area was highest was selected as the collision energy for the congener.

To determine each XPAH congener, two groups of precursor ions and respective product ions were selected for qualification and quantification, respectively (Table 1). For most substances, the peak intensities of the molecular ions ([M]^-^ and [M+2]^-^) detected by the first quadrupole were always the highest. Therefore, the molecular ions were selected as the precursor ions. Those fragments of the precursor ions showing the highest intensities (under EI mode) were selected as the product ions. The product ions were always the fragments of molecules which lost one or two halogen atoms. The gradients of the collision energies were set as 20, 30, 40, and 50 eV. We chose the final collision energies also based on the peak areas. That is, energies under which peak areas were highest for each XPAH congener were selected (Table 1). For example, peak areas of 3-ClPhe were highest under 30 eV; therefore, the collision energy for 3-ClPhe was set to be 30 eV (Figure 2).

Seven concentration levels (CS1–CS7; 1, 5, 10, 50, 200, 500, 1000 ng/mL) of targeted XPAH standards each spiked with the same concentration of isotope labelled XPAHs (as internal standard) were used to establish the calibration curves. Linear correlations were built based on the concentrations of XPAHs and relative peak areas. The ratios of concentrations of XPAH congeners to the relative internal standards were used as the X-axis, and the ratios of areas of XPAH congeners to the relative internal standards were used as the Y-axis. In order to calculate the detection limits, we repeated the measurement of CS1 (1 ng/mL) for seven times, and 3 standard deviations were used as the instrumental detection limit. The detection limits ranged 0.03–1.24 pg for the XPAH congeners (Table S3). This was at the similar level with results of our previous study on GC-HRMS analytical method of XPAHs (Jin et al. 2017b).

For the last XPAH congeners whose reference standards were not available, e.g., 3-ClPhe, 1-ClAnt, their retention times were identified based on the previous studies (Jin et al. 2017b), while the qualification and quantification ions were based on their isomers, and the concentrations were accordingly derived using the calibration curves of the isomers. This quantification method was also described and applied in previous studies on PCNs and XPAHs (Jiang et al. 2015a; Jiang et al. 2015b; Jin et al. 2017b).

### Deposition fluxes and congener profiles of XPAHs

After the establishment of the analytical method of XPAHs, we applied this method to total deposition samples. Pretreatment of the deposition samples was performed as reported above, and the recoveries of all XPAHs ranged from 67 to 121%. Field blanks were collected and analyzed together with the samples. In field blanks, 9-/2-ClPhe and 7-ClBaA were the only congeners detected. Results are presented as corrected for these blank values.

**Figure. 3:**
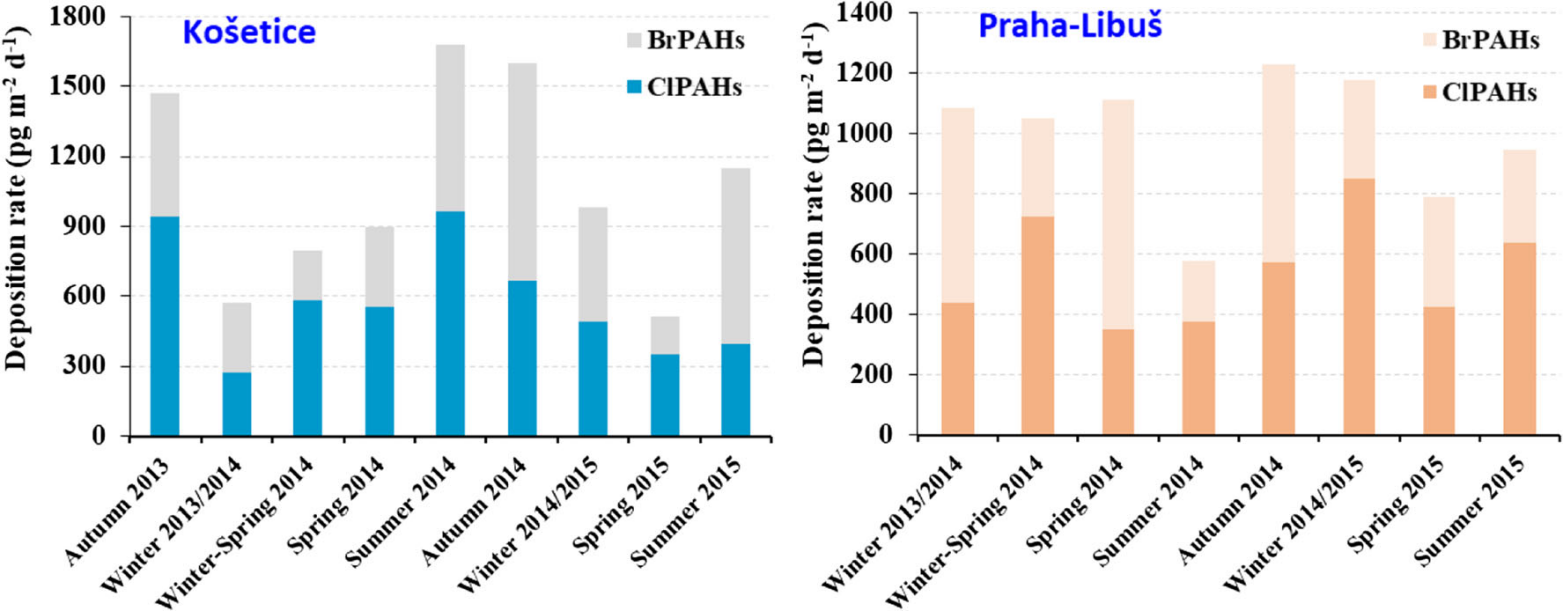
Seasonal variations of deposition fluxes of ClPAHs and BrPAHs in Košetice and Praha-Libuš.

Deposition fluxes of ClPAHs and BrPAHs were 580 (272–962) and 494 (161–936) pg m^−2^ day^−1^ in KOS, respectively, and 547 (351–724) and 449 (202–758) pg m^–2^ day^−1^ in PRA, respectively (mean (min–max); Figure 3). These ranges are similar at both sites, i.e., no gradient between semi-urban (PRA) and regional background site (KOS) is reflected. Deposition fluxes of XPAHs were reported for the first time; therefore, there is no comparison to other sites in the region or elsewhere. When compared with other POPs determined in the same samples collected in KOS, the deposition fluxes of XPAHs were at a similar range with PCBs 362 (99–2000 pg m^−2^ day^−1^), and more than 100 times lower than PAHs (110 (41–280) ng m^−2^ day^−1^ for ∑_15_PAHs; Nežiková et al. 2019). Interestingly, no significant difference between ClPAH and BrPAH deposition fluxes is found. This result is quite different from previous studies, which reported that the concentrations of ClPAHs were one order of magnitude higher than BrPAHs in air samples (Jin et al. 2017a; Jin et al. 2017c). One possible reason could be that the type and number of BrPAH congeners analyzed in this study are not the same as previous studies. For example, 2,7-Br_2_Phe contributed significantly to the total BrPAH concentrations, but were found in this study for the first time ever in samples from the atmospheric environment. Another reason could be that the sources of ClPAHs and BrPAHs were different between this study and previous studies, which focused on megacities, such as Beijing.

XPAHs’ toxic equivalencies relative to benzo[a]pyrene (BaP_eq_ concentrations) were calculated following previous studies (Jin et al. 2020b; Ohura et al. 2007; Ohura et al. 2009). BaP_eq_ concentrations of ClPAHs and BrPAHs were 273 (186–459) and 56.1 (7.97–261) pg m^−2^ day^−1^ in KOS, respectively, and 260 (154–454), and 50.8 (20.5–120) pg m^−2^ day^−1^ in PRA, respectively. On average, ClPAHs contributed more than 80% of the total BaP_eq_ concentrations in both KOS and PRA, which is more than ClPAHs’ contribution in units of mass concentration.

**Figure. 4:**
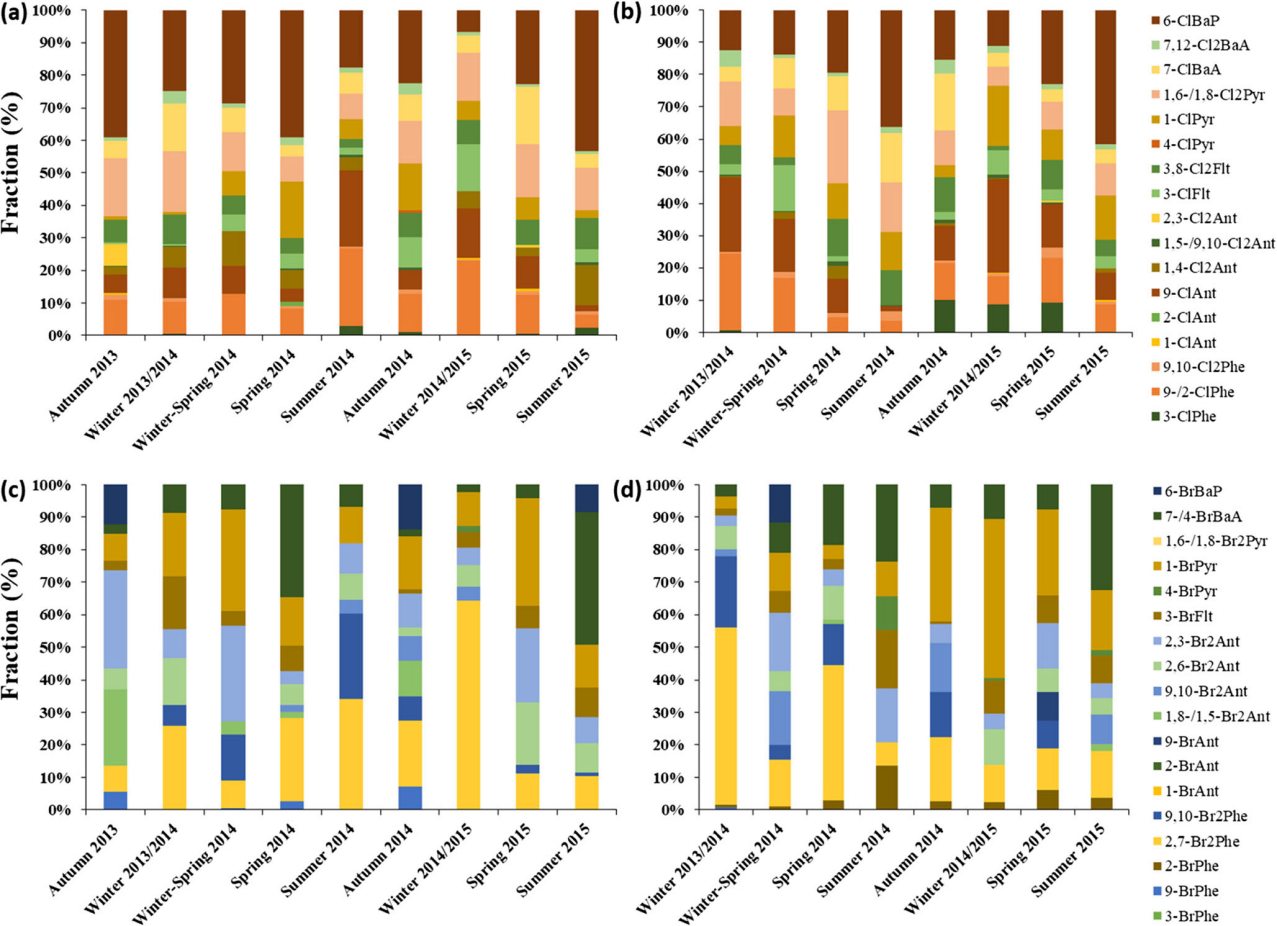
Congener distributions of (a, b) ClPAHs and (c, d) BrPAHs in total deposition samples collected in (a, c) Košetice and (b, d) Praha-Libuš.

The congener distributions of XPAHs in the total deposition samples collected from KOS and PRA are shown in Figure 4. The dominant ClPAH congeners for the total deposition samples in both KOS and PRA are 6-ClBaP, 1-ClPyr, 9-ClAnt, and 9-/2-ClPhe. Dominance of the high molecular weight congeners, such as 6-ClBaP in the deposition samples, is consistent with those in the particulate samples in days of the non-heating seasons in Beijing (Jin et al. 2017a; Jin et al. 2018). Dominant BrPAH congeners were 1-BrPyr, 2,7-Br_2_Phe, 9,10-Br_2_Phe, 2,3-Br_2_Ant, and 7-/4-BrBaA. This is different from the air samples collected in Beijing, which reported the dominance of 1-BrPyr, 1,8-/1,5-Br_2_Ant, and 1-BrAnt. This discrepancy points to different BrPAH sources in central Europe as compared to Beijing.

### Seasonal variations and distributions between particulate and dissolved depositions

Seasonal variations of XPAH deposition fluxes are shown in Figure 3. In KOS, the highest deposition flux of XPAHs was observed in summer/autumn, while the lowest fluxes were observed in winter. Hereby, the BrPAH’s summer/autumn maximum was somewhat more pronounced than the ClPAHs’. No clear seasonal such trend is found in PRA. There, the highest ClPAH fluxes were observed in winter. Among congener distributions, the percentage of 6-ClBaP increased in summer/autumn samples at both sites. No other consistent seasonal signal of congeners was observed. This is quite different from previous studies of XPAH in aerosol particles, which found that the percentage of 6-ClBaP increased significantly in winter (Jin et al. 2017a; Ohura et al. 2008; Ohura et al. 2016). Meteorological conditions and emission sources might have influenced these patterns in deposition. BaP_eq_ concentrations of XPAHs in KOS and PRA showed similar tendencies; i.e., the concentrations were higher in summer to autumn than in spring (Figure S1).

**Figure. 5:**
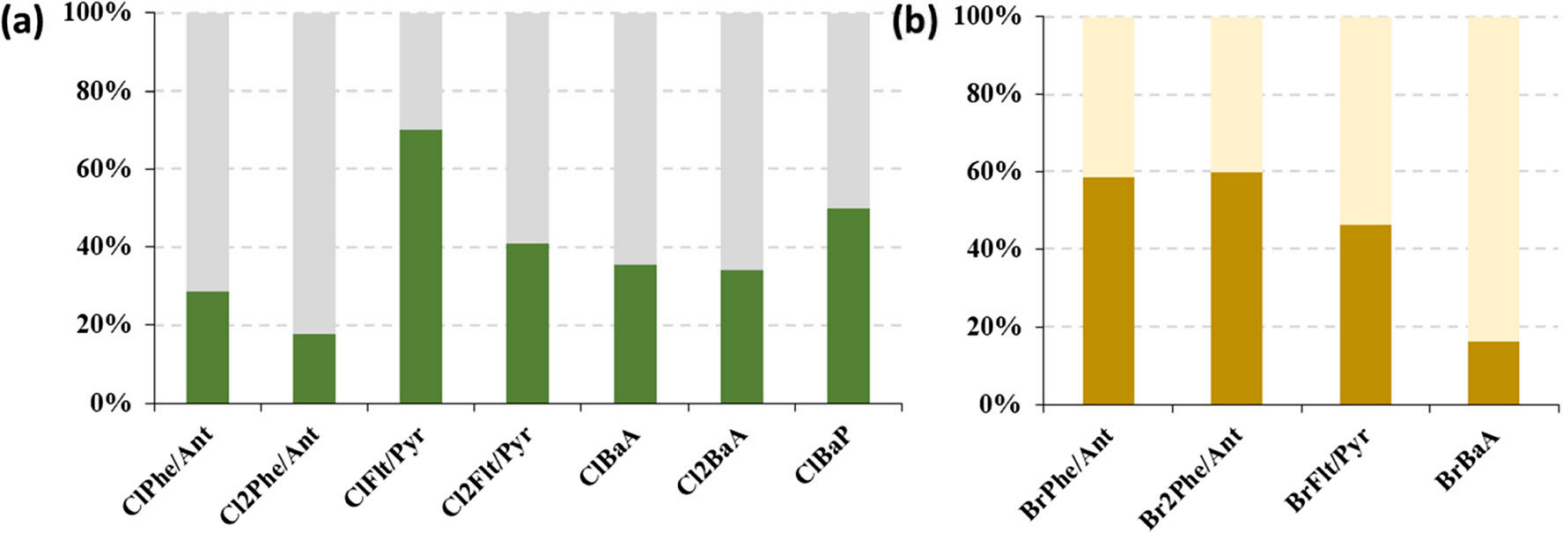
Average mass distributions between dissolved (light colors) and particulate (dark colors) phases of (a) ClPAHs and (b) BrPAHs at both sites.

Distributions of XPAHs across dissolved (XAD) and particulate (filters) phases (particulate mass fraction *θ*_*dep*_) are shown in Figure S2. There is no obvious tendency or temporal trend of *θ*_*dep*_. The *θ*_*dep*_ of ClPAHs and BrPAHs were 50.0 and 56.4%, respectively, in KOS, and 55.9 and 48.1 %, respectively, in PRA. Average phase distributions of ClPAH and BrPAH homologues in the deposition samples are shown in Figure 5. Only the homologues whose detection rate were higher than 80% in both particulate phase and dissolved phase of samples were included. For ClPAHs, by average, the *θ*_*dep*_ of the high molecular weight ClPAH homologues, such as ClFlt/Pyr and ClBaP, were higher than those of the low molecular weight ClPAH homologues, such as ClPhe/Ant. This is consistent with the vapor pressures and the gas-particle partitioning of XPAHs in air (Jin et al. 2017c). For BrPAHs, however, it is found that the *θ*_*dep*_ values for high molecular weight homologues, such as BrFlt/Pyr and BrBaA, were lower than those of the lighter homologues, such as BrPhe/Ant. Unlike the Cl-C bond, the Br-C bond of aromatic C can be subject to direct photodegradation (Wan et al. 2018), photolysis of BrPAHs in condensed phase but not in the gas-phase, as observed for polybrominated diphenyl ethers (Geller et al. 2008; Söderström et al. 2004), but not for chlorinated aromatics could be a possible explanation for this trend.

## Conclusions

In this study, we established a GC-MS/MS analytical method for the determination of XPAHs in total deposition samples collected in central Europe. Recoveries (65–126%) and detection limits (<1 pg) for most targeted congeners were at similar levels with the previous GC-HRMS method. This makes the newly optimized method suitable for determination of ultra-trace XPAH amounts as anticipated in samples of atmospheric origin from moderately polluted or even remote environments. XPAHs in the European atmospheric environment were reported for the first time, i.e., in deposition samples from central Europe, along with seasonal variations and the distributions of XPAHs between particulate and dissolved depositions. No urban-rural gradient is reflected for these pollutants, possibly related to their long atmospheric residence time, hence, homogeneous distribution in air. The XPAH distribution, their main, supposedly industrial sources in Europe and the related congener patterns should be studied in order to understand the atmospheric cycling of this “emerging” class of pollutants.

## Supplementary information

ESM 1 (DOCX 127 kb)
