# Supplementary material for: Atmospheric deposition of chlorinated and brominated polycyclic aromatic hydrocarbons in central Europe analyzed by GC-MS/MS
Source: Environ Sci Pollut Res Int. 2021 Jun 26;28(43):61360–8. doi: 10.1007/s11356-021-15038-3 (PMC8580896; doi:10.1007/s11356-021-15038-3)
Supplement: Supplementary file 1 — (DOCX 127 kb) [file 11356_2021_15038_MOESM1_ESM.docx]

***→→Supporting information for***

**Atmospheric deposition of chlorinated and brominated polycyclic aromatic hydrocarbons in central Europe analyzed by GC-MS/MS**

Rong Jin^1,2^, Benjamin A. Musa Bandowe^2^, Minghui Zheng^1,3^, Guorui Liu^1,3^, Barbora Nežiková^4^, Roman Prokeš^4^, Pavel Čupr^4^, Jana Klánová^4^, Gerhard Lammel^2,4*^

^1^ School of Environment, Hangzhou Institute for Advanced Study, University of Chinese Academy of Sciences, 310024, Hangzhou, China

^2^ Multiphase Chemistry Department, Max-Planck Institute for Chemistry, Hahn-Meitner-Weg 1, 55128 Mainz, Germany

^3^ State Key Laboratory of Environmental Chemistry and Ecotoxicology, Research Centre for Eco-environmental Sciences, Chinese Academy of Sciences, 100085, Beijing, China

^4^ Research Centre for Toxic Compounds in the Environment, Masaryk University, Kamenice 5, 62500 Brno, Czech Republic

**Table and figure lists:**

Table S1. Sampling periods of this study.

Table S2. Sequences of solvents run for clean-up of total deposition samples.

Table S3. Absolute detection limits (pg) for XPAHs.

Figure S1. Seasonal variations of BaP equivalent deposition fluxes of ClPAHs and BrPAHs in Košetice and Praha-Libuš.

Figure S2. Distributions of ClPAHs (a,b) and BrPAHs (c,d) between XAD (dark) and filters (light) in samples collected in Košetice: (a,c) and Praha-Libuš: (b,d)

Table S1. Sampling periods of this study.

| **Sampling period** |  | **Košetice** | **Praha-Libuš** |
| --- | --- | --- | --- |
| 2013.7.31-2013.10.24 | Autumn 2013 | √ |  |
| 2013.10.24-2014.1.16 | Winter 2013/2014 | √ | √ |
| 2014.1.16-2014.4.10 | Winter-Spring 2014 | √ | √ |
| 2014.4.10-2014.7.13 | Spring 2014 | √ | √ |
| 2014.7.13-2014.9.25 | Summer 2014 | √ | √ |
| 2014.9.25-2014.12.18 | Autumn 2014 | √ | √ |
| 2014.12.18-2015.3.12 | Winter 2014/2015 | √ | √ |
| 2015.3.12-2015.6.4 | Spring 2015 | √ | √ |
| 2015.6.4-2015.8.27 | Summer 2015 | √ | √ |

Table S2. Sequences of solvents run for clean-up of total deposition samples.

| Testing sequence | 1^st^ elution | 2^nd^ elution | 3^rd^ elution |
| --- | --- | --- | --- |
| No.1 | Mix^a^ | Mix^a^ | Mix^a^ |
| No.2 | Mix^a^ | DCM^b^ | EA^c^ |
| No.3 | Mix^a^ | EA^c^ | DCM^b^ |

^a^ 10 mL hexane: dichloromethane= 4:1V/V

^b^ 10 mL dichloromethane (DCM)

^c^ 10 mL ethyl acetate (EA)

Table S3. Absolute detection limits (pg) for XPAHs.

| No. | Name | Acronym | Detection limit | No. | Name | Acronym | Detection limit |
| --- | --- | --- | --- | --- | --- | --- | --- |
| 1 | 1-chloroanthracene | 1-ClAnt | 0.65 | 22/23 | 1,8-dibromoanthracene/  1,5-dibromoanthracene | 1,8-/1,5-Br_2_Ant | 0.61 |
| 2 | 2-chloroanthracene | 2-ClAnt | 0.65 | 24 | 9,10-dibromoanthracene | 9,10-Br_2_Ant | 0.67 |
| 3 | 3-chlorophenanthrene | 3-ClPhe | 0.65 | 25 | 2,7-dibromophenanthrene | 2,7-Br_2_Phe | 1.21 |
| 4 | 9-chloroanthracene | 9-ClAnt | 0.65 | 26 | 9,10-dibromophenanthrene | 9,10-Br_2_Phe | 0.58 |
| 5/6 | 9-chlorophenanthrene/  2-chlorophenanthrene | 9-/2-ClPhe | 0.65 | 27 | 1-bromopyrene | 1-BrPyr | 1.24 |
| 7 | 2-bromophenanthrene | 2-BrPhe | 0.28 | 28 | 4-bromopyrene | 4-BrPyr | 1.19 |
| 8 | 3-bromophenanthrene | 3-BrPhe | 0.45 | 29 | 2,3-dibromoanthracene | 2,3-Br_2_Ant | 0.53 |
| 9 | 9-bromophenanthrene | 9-BrPhe | 0.42 | 30 | 2,6-dibromoanthracene | 2,6-Br_2_Ant | 0.52 |
| 10 | 1-bromoanthracene | 1-BrAnt | 0.31 | 31/32 | 1,6-dichloropyrene/1,8-dichloropyrene | 1,6-/1,8-Cl_2_Pyr | 0.72 |
| 11 | 2-bromoanthracene | 2-BrAnt | 0.33 | 33 | 3,8-dichlorofluoranthene | 3,8-Cl_2_Flt | 0.37 |
| 12 | 9-bromoanthracene | 9-BrAnt | 0.45 | 34 | 7-chlorobenz[a]anthracene | 7-ClBaA | 0.23 |
| 13 | 1,4-dichloroanthracene | 1,4-Cl_2_Ant | 0.27 | 35/36 | 1,6-dibromopyrene/1,8-dibromopyrene | 1,6-/1,8-Br_2_Pyr | 1.80 |
| 14/15 | 1,5-dichloroanthracene/  9,10-dichloroanthracene | 1,5-/9,10-Cl_2_Ant | 0.33 | 37/38 | 7-bromobenz[a]anthracene/  4-bromobenz[a]anthracene | 7-/4-BrBaA | 0.46 |
| 16 | 9,10-dichlorophenanthrene | 9,10-Cl_2_Phe | 0.15 | 39 | 7,12-dichlorobenz[a]anthracene | 7,12-Cl_2_BaA | 0.20 |
| 17 | 2,3-dichloroanthracene | 2,3-Cl_2_Ant | 0.32 | 40 | 6-chlorobenzo[a]pyrene | 6-ClBaP | 0.03 |
| 18 | 1-chloropyrene | 1-ClPry | 0.17 | 41 | 6-bromobenzo[a]pyrene | 6-BrBaP | 0.55 |
| 19 | 3-chlorofluoranthene | 3-ClFlt | 0.18 |  |  |  |  |
| 20 | 4-chloropyrene | 4-ClPyr | 0.18 |  |  |  |  |
| 21 | 3-bromofluoranthene | 3-BrFlt | 0.46 |  |  |  |  |


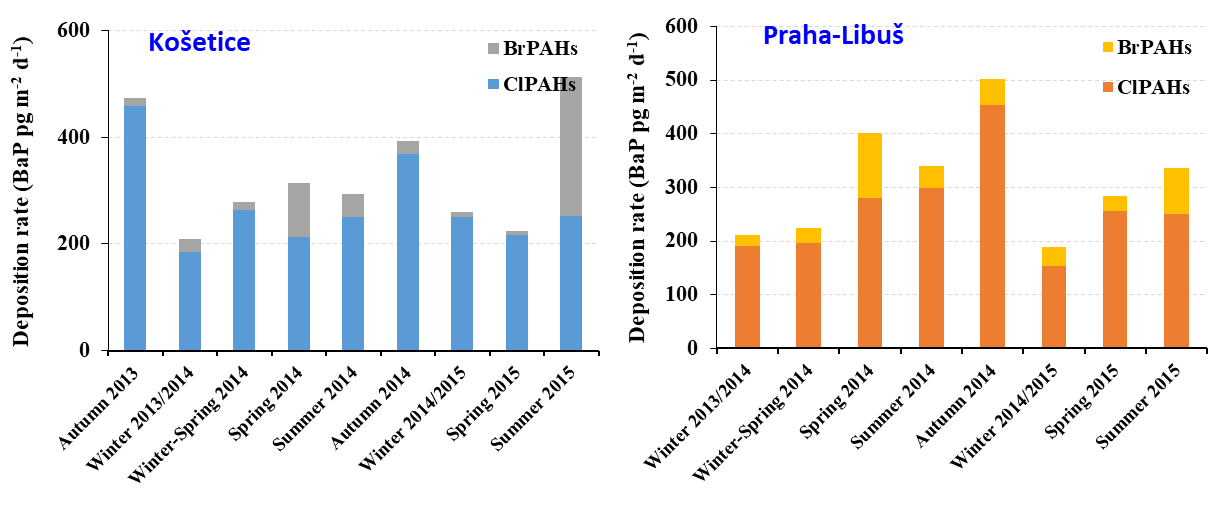


Figure S1. Seasonal variations of BaP equivalent deposition fluxes of ClPAHs and BrPAHs in Košetice and Praha-Libuš.


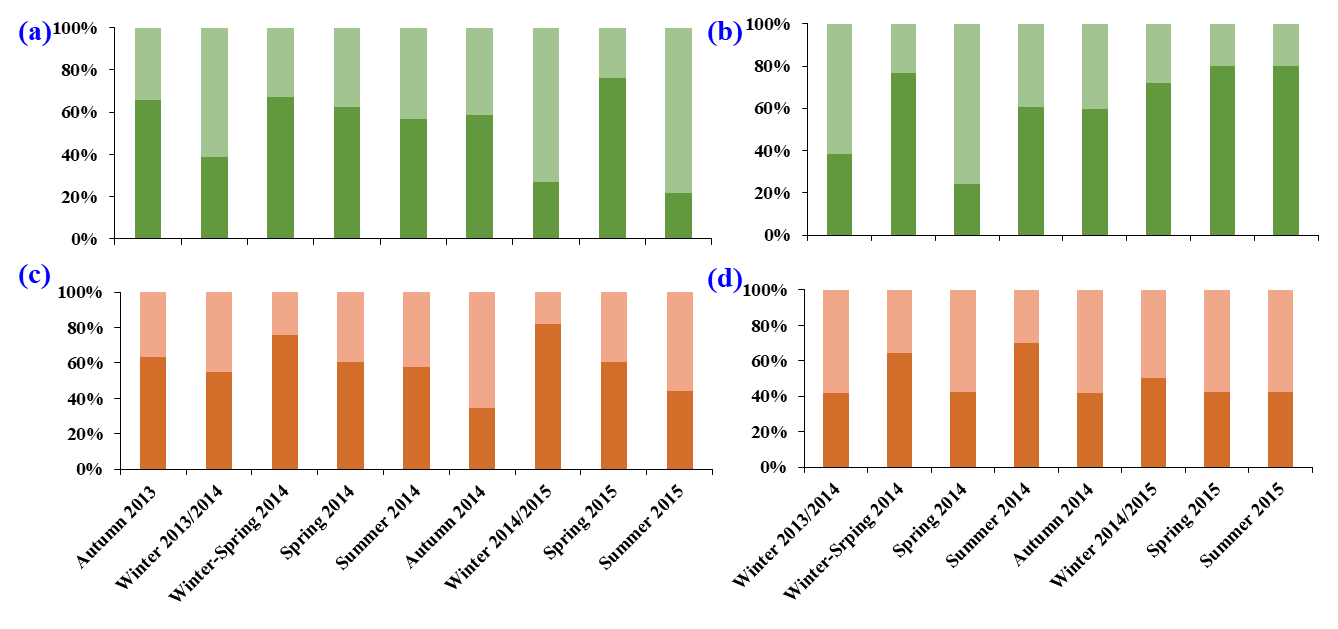


Figure S2. Distributions of ClPAHs (a,b) and BrPAHs (c,d) between XAD (dark) and filters (light) in samples collected in Košetice: (a,c) and Praha-Libuš: (b,d)
